# Determination of optimum intensity and duration of exercise based on the immune system response using a machine-learning model

**DOI:** 10.1038/s41598-023-34974-3

**Published:** 2023-05-22

**Authors:** Shirin Asadi, Bakhtyar Tartibian, Mohammad Ali Moni

**Affiliations:** 1 Department of Exercise Physiology, Faculty of Physical Education and Sport Sciences, Allameh Tabataba’i University, Tehran, Iran; 2 Department of Exercise Physiology, Faculty of Physical Education and Sports Sciences, Allameh Tabataba’i University, Tehran, Iran; 3 Artificial Intelligence and Data Science, Faculty of Health and Behavioural Sciences, School of Health and Rehabilitation Sciences, The University of Queensland, Brisbane, Australia

**Keywords:** Immunology, Machine learning, Disease prevention

## Abstract

One of the important concerns in the field of exercise immunology is determining the appropriate intensity and duration of exercise to prevent suppression of the immune system. Adopting a reliable approach to predict the number of white blood cells (WBCs) during exercise can help to identify the appropriate intensity and duration. Therefore, this study was designed to predict leukocyte levels during exercise with the application of a machine-learning model. We used a random forest (RF) model to predict the number of lymphocytes (LYMPH), neutrophils (NEU), monocytes (MON), eosinophils, basophils, and WBC. Intensity and duration of exercise, WBCs values before exercise training, body mass index (BMI), and maximal aerobic capacity (VO_2_ max) were used as inputs and WBCs values after exercise training were assessed as outputs of the RF model. In this study, the data was collected from 200 eligible people and K-fold cross-validation was used to train and test the model. Finally, model efficiency was assessed using standard statistics (root mean square error (RMSE), mean absolute error (MAE), relative absolute error (RAE), root relative square error (RRSE), coefficient of determination (R^2^), and Nash–Sutcliffe efficiency coefficient (NSE)). Our findings revealed that the RF model performed well for predicting the number of WBC with RMSE = 0.94, MAE = 0.76, RAE = 48.54, RRSE = 48.17, NSE = 0.76, and R^2^ = 0.77. Furthermore, the results showed that intensity and duration of exercise are more effective parameters than BMI and VO_2_ max to predict the number of LYMPH, NEU, MON, and WBC during exercise. Totally, this study developed a novel approach based on the RF model using the relevant and accessible variables to predict WBCs during exercise. The proposed method can be applied as a promising and cost-effective tool for determining the correct intensity and duration of exercise in healthy people according to the body’s immune system response.

## Introduction

The immune system is a complex interaction between cells and molecules that has a variety of functions to protect the host against possible microorganism invasions and prevent diseases^1^. Various factors affect the functioning of the immune system; exercise is one of these factors. Many studies have proposed exercise as a tool to study the interactions between metabolic stress and the immune system and have used sportmics approach to understand exercise-induced cellular and metabolic modifications^2–5^. A review of previous studies showed that although health promotion is one of the implications of exercise, exercise with unsuitable intensity and duration weaken the immune system and increases the risk of contracting various diseases^6,7^. Also, the various findings related to the impact of exercise on the immune system showed that in addition to the intensity and duration of exercise training, different factors such as gender^8–10^, age, physical fitness level and body mass index (BMI)^11^, body fat percentage^12^ affect white blood cells (WBCs) level. Although there has been considerable research on the interaction between exercise and the immune system (WBCs), still a comprehensive result or a specific pattern has not been provided related to the immune system response to different intensities and durations of exercise, because this relationship is nonlinear and much complicated^13^. Therefore, until now, researchers have not been able to achieve the optimal pattern of exercise for people, while the discovery of this pattern is vital because exercise with proper intensity and duration can boost the immune system and reduce the chance of contracting diseases such as viral infections, cancer, and inflammatory diseases^7^.

In recent years, machine learning (ML) models have been increasingly noticed as new technology and a powerful tool in information processing, prediction, and modelling^14–19^. Decision-tree (DT) algorithms are one of the main tools of ML that have been used in a wide spectrum of applications in clinical fields, including the diagnosis and prediction of cardiovascular diseases and cancers^20^. Random forest (RF) is the most successful general-purpose algorithm in modern times^21^ that has shown the highest accuracy among different variants of supervised ML algorithms in most clinical studies^20^. ML algorithms can be divided into three categories according to the way the machine is being taught: supervised, unsupervised and semi-supervised. Supervised ML algorithms are based on response variables that can supervise the analysis^20,22^.

Despite the increased use of intelligent techniques for medical decision support systems^23^, there are very few studies in the area of exercise immunology^24,25^. Furthermore, to the best of our knowledge, there are no studies that have used ML models (e.g., RF) to develop an efficient tool to predict the number of WBCs during exercise. Thus, we provided a novel approach based on the RF model to predict the number of lymphocyte (LYMPH), neutrophil (NEU), monocyte (MON), eosinophil (EOS), basophil (BASO) and WBC during exercise for healthy people. Our proposed method is easily applicable with the least limitations in applying different factors. In this regard, the present study has two main objectives: (1) investigate an RF model to predict the number of WBCs during exercise and (2) investigate the importance of intensity and duration of exercise in the prediction of the number of WBCs during exercise.

## Methods

### Subjects

This study involved human participants and was approved by the Research Ethics Committee and all methods were performed in accordance with the relevant regulations. The objectives and the research process were clearly explained to all of the subjects, and all participants provided written consent prior to the start of the study. A total of 200 eligible healthy subjects (100 men, 50.0%) in the age range of 18–60 years participated in this study. For knowing of health history (e.g., the presence of infectious, cardiovascular, inflammatory or immune diseases), subjects were screened with questionnaire before the study period. Also, the participants were asked not to take anti-inflammatory agents, steroids and vitamin supplements for 2 weeks before the exercise sessions and refrain from exercise training or vigorous physical activity. The statistical information of 200 individuals is summarised in Table 1.

**Table 1:** characteristics of participants and input and output data.

Feature	Total data	
	Min–Max	Mean ± SD
Age (years)	18–60	36.54 ± 10.86
Weight (kg)	49–130	76.09 ± 12.83
Height (cm)	154–191	169 ± 9
BMI (kg.m^-2^)	18.44–40.12	26.65 ± 4.38
VO_2_ max (ml.kg^-1^. Min^-1^)	21.1–55.74	37.03 ± 7.63
HR_target1_ (bpm)	116–175	141.93 ± 14.19
HR_target2_ (bpm)	131–189	155.57 ± 15.88
Duration (min)	1–138	46.19 ± 38.23
WBC_1_ (10^3^/mm^3^)	4.25–10.32	7.14 ± 1.52
WBC_2_ (10^3^/mm^3^)	5.36–14.23	8.90 ± 1.96
LYMPH_1_ (10^3^/mm^3^)	1.01–4.71	2.42 ± 0.70
LYMPH_2_ (10^3^/mm^3^)	0.86–6.86	3.32 ± 1.22
NEU_1_ (10^3^/mm^3^)	1.65–6.48	3.76 ± 1.15
NEU_2_ (10^3^/mm^3^)	1.85–8.73	4.63 ± 1.39
MON_1_(10^3^/mm^3^)	0.28–1.00	0.56 ± 0.15
MON_2_ (10^3^/mm^3^)	0.22–1.30	0.74 ± 0.21
EOS_1_(10^3^/mm^3^)	0.01–0.80	0.23 ± 0.17
EOS_2_ (10^3^/mm^3^)	0.01–1.07	0.26 ± 0.20
BASO_1_(10^3^/mm^3^)	0.00–0.08	0.03 ± 0.02
BASO_2_ (10^3^/mm^3^)	0.00–0.22	0.05 ± 0.03

BMI = body mass index. VO_2_ max = maximal aerobic capacity. HR_target1_ = the minimum of target heart rate of subjects in determined intensity. HR_target2_ = the maximum target heart rate of subjects in determined intensity. Duration = exercise training duration. WBC_1_ = white blood cell values before exercise training. LYMPH_1_ = lymphocyte values before exercise training_._ NEU_1_ = neutrophil values before exercise training. MON_1_ = monocyte values before exercise training. EOS_1_ = eosinophil values before exercise training. BASO_1_ = basophil values before exercise training. WBC_2_ = white blood cell values after exercise training. LYMPH_2_ = lymphocyte values after exercise training_._ NEU_2_ = neutrophil values after exercise training. MON_2_ = monocyte values after exercise training. EOS_2_ = eosinophil values after exercise training. BASO_2_ = basophil values after exercise training.

### The protocol

We measured the anthropometric indicators (weight, height and BMI) using standard techniques. To evaluate VO_2_ max, the subjects completed a Bruce test to voluntary exhaustion on a calibrated treadmill^26^ in the cardiology clinic. Given that changes in the immune system depend on exercise intensity (low, moderate, high)^27^, hence in this study, exercise protocol was planned according to the intensity suggested by the American College of Sports Medicine (ACSM) (i.e., low intensity (50–63% of HR_max_), moderate intensity (64–76% of HR_max_), and high intensity (77–93% of HR_max_))^28^. Before implementing the exercise session, the maximum heart rate (HR_max_) using the Tanaka method was computed^29^. Then, the minimum and maximum target heart rate (HR_target_) based on the determined intensity for each subject was obtained by the Karvonen method^30^.

The participants performed on a treadmill (Rodby, RL1602E, Sweden) the exercise protocol in accordance with the determined HR_target_ (i.e., between the minimum and maximum HR_target_). The heart rate of the subjects during exercise protocol was monitored continuously with a Polar watch and chest strap (Polar Electro Oy, Kempele, Finland) to ensure that the exercise program was performed according to the intensity specified by ACSM. It is noteworthy that subjects were tested in an individual training condition in a public fitness centre, and for each subject only one of the above-mentioned intensities has performed. The duration of exercise training according to the capacity of the subjects was considered, hence a certain duration was not determined for subjects in advance. The individual’s capacity is influenced by different factors such as age, gender, BMI, and intensity of exercise^31^. Blood samples (3 ml of peripheral venous blood) were taken at baseline and immediately after the completion of the exercise to determine plasma levels of leukocytes. Finally, the collected data were used for input and output of the RF model to predict the WBCs level.

### Random forest (RF)

RF as DT- based algorithm is an extremely successful classification and regression method. This approach, aside from having few parameters to tune, is generally recognized for its accuracy and its ability to deal with small sample sizes^32^. The approach combines several randomized decision trees and produces a forest of decision trees. Every tree predicts a class which the final decision was achieved by averaging all predictions^19^. It is necessary to mention, the data before the modelling process was transformed to range from 0 to 1 because the normalization of data minimizes bias and ensures that they receive the same attention within the network^33^ In WBCs modelling, to avoid over-fitting, K-fold cross-validation was applied to train and test the RF model. In this approach, the whole dataset was randomly partitioned into 5 equal sized subsamples (40 cases). Of the five subsamples, four samples for training (160 cases) and one sample for testing (40 cases) were used. this process repeated 5 times, in each time one of the subsamples was used as the validation data^19^.

### Model structure and features importance

The use of proper input vectors in supervised ML algorithms is important in the modelling process^34^. In this simple prediction model, the effective factors on WBCs based on past studies, including BMI, VO_2_ max, intensity (HR_target1_ and HR_target2_) and duration of exercise training for input was adopted. We also considered WBCs values before exercise training as a required input because the number of WBCs differs between individuals. For the model output, the number of WBCs after exercise training was assessed and finally, 6 different scenarios were established for modelling according to Table 2.

**Table 2:** Input and output scenarios.

Scenario number	Inputs	Output
1	${\text{WBC}}{1}+\text{BMI}+ {\text{VO}}{2}\max\,+{\text{HR}} {\text{target}}{1}+ {\text{HR}} {\text{target}}{2}+ {\text{Duration}}$	${\text{WBC}}{2}$
2	${\text{LYMPH}}{1}+ \text{BMI}+ {\text{VO}}{2}\max\,+ {\text{HR}} {\text{target}}{1}+ \text{HR} {\text{target}}{2}+ {\text{Duration}}$	${\text{LYMPH}}{2}$
3	${\text{NEU}}{1}+\text{BMI}+ {\text{VO}}{2}\max+ {\text{HR}} {\text{target}}{1}+ {\text{ HR}} {\text{target}}{2}+ {\text{Duration}}$	${\text{NEU}}{2}$
4	${\text{MON}}{1}+\text{ BMI}+ {\text{VO}}{2}\max\,+{\text{HR}} {\text{target}}{1}+ {\text{HR}} {\text{target}}{2}+ {\text{Duration}}$	${\text{MON}}{2}$
5	${\text{EOS}}{1}+\text{BMI}+{\text{VO}}{2}\max\,+{\text{HR}} {\text{target}}{1}+ {\text{HR}} {\text{target}}{2}+{\text{Duration}}$	${\text{EOS}}{2}$
6	${\text{BASO}}{1}+\text{BMI}+ {\text{VO}}{2}\max\,+{\text{HR}} {\text{target}}{1}+ {\text{HR}} {\text{target}}{2}+{\text{Duration}}$	${\text{BASO}}{2}$

Feature importance due to their simplicity and interpretability of feature ranking is an important and widely used analysis method in modelling with the machine learning algorithms. Most of the supervised ML algorithms including RF provide feature importance^19^. In this study, importance of each parameter based mean decrease in impurity (MDI) was estimated.

### Evaluation criteria

Six quantitative metrics, including the Pearson coefficient of determination (R^2^), root mean squared error (RMSE), mean absolute error (MAE), relative absolute error (RAE), root relative square error (RRSE)^34^, and Nash–Sutcliffe efficiency coefficient (NSE) were used for performance analysis of the model in the testing dataset. It’s worth noting that the NSE has been used for the performance evaluation of ML models in different fields (e.g., hydrology, physics)^33,35–37^ and has been confirmed as a more reliable efficiency index compared with R^2^^33^. Therefore, we suggested it for evaluation of the results of this study. The equations for the above-mentioned indices are expressed as follows:

1 $$ {\text{RMSE}} = \sqrt {\frac{1}{n}\sum\nolimits_{i = 1}^{n} {(O_{i} - P_{i} )^{2} } } $$

2 $$ {\text{MAE}} = \frac{{\mathop \sum \nolimits_{{\text{i} = 1}}^{{\text{n}}} \left| {{\text{O}}_{{\text{i}}} - {\text{P}}_{{\text{i}}} } \right|}}{{\text{n}}} $$

3 $$ {\text{NSE = 1}} - \frac{{\mathop \sum \nolimits_{{\text{i} = 1}}^{{\text{n}}} \left( {{\text{O}}_{{\text{i}}} - {\text{P}}_{{\text{i}}} } \right)^{{2}} }}{{\mathop \sum \nolimits_{{\text{i} = 1}}^{{\text{n}}} \left( {{\text{O}}_{{\text{i}}} - {\overline{\text{O}}}} \right)^{{2}} }} $$

4 $$ {\text{R}}^{{2}} = \left[ {\frac{{\mathop \sum \nolimits_{{\text{i = 1}}}^{{\text{n}}} \left( {{\text{O}}_{{\text{i}}} - {\overline{\text{O}}}} \right)\left( {P_{{\text{i}}} - \overline{P} } \right)}}{{\sqrt {\mathop \sum \nolimits_{{\text{i = 1}}}^{{\text{n}}} \left( {{\text{O}}_{{\text{i}}} - {\overline{\text{O}}}} \right)^{{2}} } \sqrt {\mathop \sum \nolimits_{{\text{i = 1}}}^{{\text{n}}} \left( {P_{{\text{i}}} - \overline{P} } \right)^{{2}} } }}} \right]^{{2}} $$

5 $$ RAE = \frac{{\sum\nolimits_{i = 1}^{n} {\left| {O_{i} - P_{i} } \right|} }}{{\sum\nolimits_{i = 1}^{n} {\left| {O_{i} - \overline{O} } \right|} }} $$

6 $$ RRSE = \sqrt {\frac{{\sum\nolimits_{i = 1}^{n} {\left( {O_{i} - P_{i} } \right)^{2} } }}{{\sum\nolimits_{i = 1}^{n} {\left( {O_{i} - \overline{O} } \right)^{2} } }}} $$

where n is the number of data, O_i_ and P_i_ are the ith actual and predicted values, respectively. Also, $\underline{O}$ and $\underline{P}$ are the average of actual and predicted values, respectively. RMSE and MAE range from 0 to + ∞, NSE ranges from − ∞ to 1, and R^2^ ranges from 0 to 1which higher NSE and R^2^ values and lower RMSE and MAE values indicate better efficiency of models^33,34^.

### Ethics approval

This study involved human participants and was approved by the Research Ethics Committee of Allameh Tabataba'i University (reference number IR.ATU.REC.1401.052). Participants gave informed consent to participate in the study before taking part.

## Results

### Result of scenarios analysis

The RF model to predict the number of WBCs was evaluated using performance indices (RMSE, MAE, RAE, RRSE, NSE, and R^2^). Their values for all scenarios during the testing phase are shown in Table 3.

**Table 3:** Performance of the RF model for prediction of WBCs levels.

Scenario number	RMSE (10^3^/mm^3^)	MAE (10^3^/mm^3^)	RAE (%)	RRSE (%)	NSE	R^2^
1	0.94	0.76	48.54	48.17	0.76	0.77
2	0.74	0.57	50.82	53.23	0.71	0.72
3	0.69	0.55	58.30	56.90	0.67	0.68
4	0.12	0.08	51.39	58.02	0.66	0.66
5	0.11	0.07	50.49	56.41	0.68	0.69
6	0.03	0.02	88.09	94.94	0.09	0.11

R^2^ = Pearson coefficient of determination. RMSE = Root mean squared error. MAE = Mean absolute error. NSE = Nash–Sutcliffe efficiency coefficient. RAE = relative absolute error. RRSE = root relative square error.

### Result of feature importance analysis

**Figure 1:**
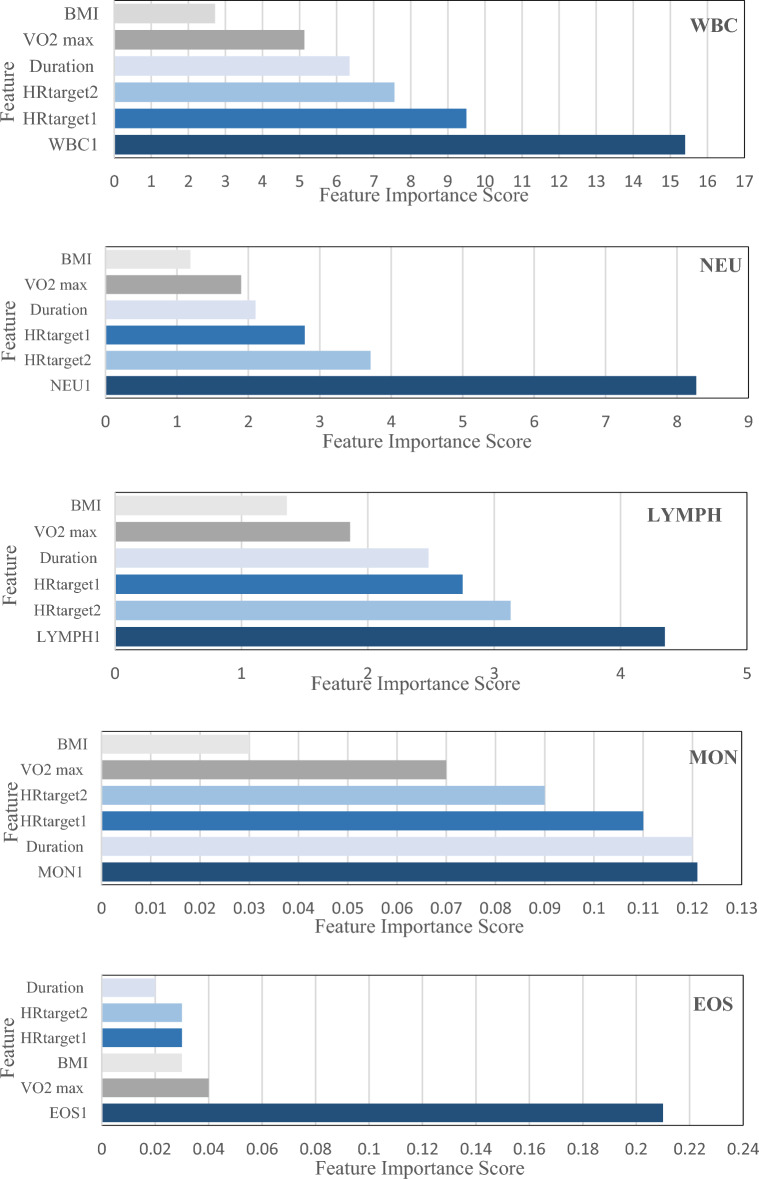
Graphical representation of features importance.

We also estimated the features importance in all the scenarios. The results of the features importance score are indicated in Table 4 and graphically in Fig. 1.

**Table 4:** Features importance based on MDI for prediction of WBCs levels.

Features name	Scenario number					
	1	2	3	4	5	6
HR_target1_	9.50	2.79	2.75	0.11	0.03	0.00
HR_target2_	7.56	3.71	3.13	0.09	0.03	0.00
Duration	6.35	2.10	2.48	0.12	0.02	0.00
VO_2_ max	5.13	1.90	1.86	0.07	0.04	0.00
BMI	2.72	1.19	1.36	0.03	0.03	0.00
WBC	15.40	–	–	–	–	–
NEU	–	8.27	–	–	–	–
LYM	–	–	4.35	–	–	–
MON	–	–	–	0.12	–	–
EOS	–	–	–	–	0.21	–
BAS	–	–	–	–	–	0.00

**Figure 2:**
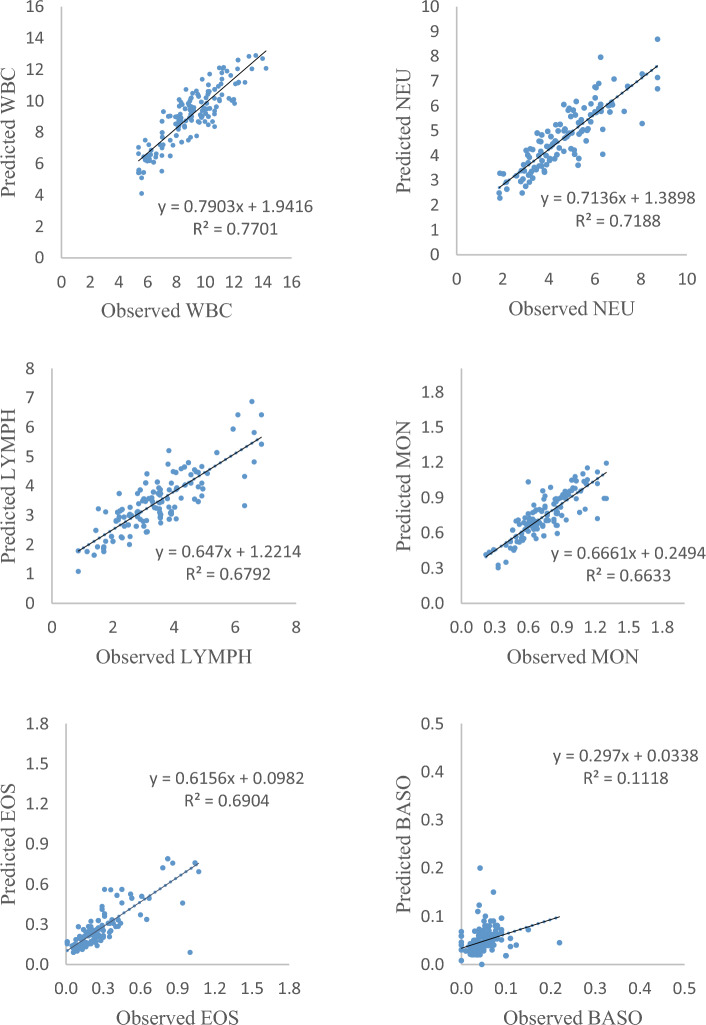
Scatter plots of the actual WBCs and predicted WBCs by RF during the testing phase.

Also, to assess the efficiency of the best scenario of the developed model, correlations between actual and predicted values of WBC, NEU, LYMPH, MON, BASO, and EOS during the testing phase were presented in (Fig. 2). Comparisons amongst all tested models showed that the model for predicting BASO (R^2^ = 0.11) had the lowest correlation and the model for predicting WBC (R^2^ = 0.77) had the best correlation and predicted WBC were in closer agreement with the actual WBC values compared with NEU, LYMPH, MON, BASO, and EOS.

**Figure 3:**
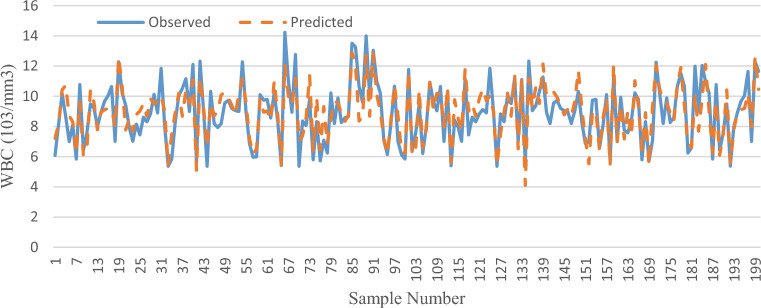
Curve of the actual WBC versus predicted WBC by RF during the testing phase.

Moreover, the plot of variations of actual values versus predicted values for the best scenario (i.e., WBC) during the testing phase was shown in Fig. 3.

## Discussion

### Evaluation of the results

Based on the obtained results, for predicting the number of WBC, LYMPH, NEU, and MON, the most effective feature was values of WBC, LYMPH, NEU, and MON before training followed by intensity and duration of exercise; for predicting the number of EOS, the most effective feature was values of EOS before training followed by VO_2_ max, and BMI; and for predicting the number of BASO, no feature was not effective. These results are consistent with the physiological function of the body. Adjustment of the immune response using the central nervous system is performed by bidirectional signals between the nervous, endocrine and immune systems^38^. Two important pathways for immune system dysregulation are: The hypothalamic–pituitary–adrenal axis and the autonomic nervous system. Exercise can activate the hypothalamic–pituitary–adrenal axis and the sympathetic nervous system which stimulates the secretion of the hormones such as catecholamines (adrenaline and noradrenaline), adrenocorticotropic hormone, and cortisol. Each of these hormones can cause quantitative and qualitative changes in immune function^39^. For example, an increase in adrenaline concentration and a lesser degree of noradrenaline are the main factors of LYMPH dynamics in acute exercise^40^. Also, some studies showed that cortisol, primarily by the demargination of cells from the blood vessel walls, with a minor contribution from the bone marrow, cause neutrophilia^41^. Most researchers in the field of exercise immunology believe that the immune system reflects the magnitude of physiological stress experienced by the exerciser^42^. Exercise-induced muscle tissue injury and inflammation elicit a strong immune response involving NEU, EOS, BASO, MON, and macrophages. Immune-specific proteins (e.g., oxylipins) are produced to modulate the innate immune response, involved in initiating, mediating, and resolving this process^43,44^. The majority of the expressed immune-related proteins (e.g., lysozyme C, neutrophil elastase and defensing1, cathelicidin antimicrobial peptide, α-actinin-1, and profilin-1) are involved with pathogen defense and immune cell chemotaxis and locomotion. Other proteins (e.g., serum amyloid A-4, myeloperoxidase, plasma protease C1 inhibitor, α-2-HS-glycoprotein, andα-1-acid glycoprotein 2) increase during recovery and affect the inflammatory acute phase response^43^. This profound, exercise-induced perturbation in metabolites, lipid mediators, and proteins likely has a direct influence on immune function and results in transient immune dysfunction^45^.

Low effectiveness of intensity and duration of exercise in the prediction of the number of EOS may be because of more effects of EOS in allergic diseases and parasitic infections^46^. Moreover, it may show that these cells need more severe stress than the stress induced in this study^47^. Also, the high impact of intensity and duration of exercise on the prediction of WBC levels considering the effect of exercise on NEU, LYMPH, and MON and a large volume of them in leukocytes (NEU (about 60%), lymphocytes (about 30%), and MON (about 5.3%)^48^), can be justifiable.

A comparison amongst different scenarios based on standard statistics (RAE, RRSE, NSE and R^2^) showed that scenario 1 to predict the number of WBC, had the highest performance, while to predict the number of BASO, the results of the RF model were not acceptable. Generally, based on the NSE metric, the RF model for predicting NEU, LYMPH, MON, and EOS levels showed good performance (0.65 < NSE ≤ 0.75) and for predicting WBC showed very good performance (0.75 < NSE ≤ 1.00)^33,49^.

The comparison of the actual versus predicted WBC graph in Fig. 3 confirms that, although there is a relatively good agreement between actual and predicted values of WBC, in some cases, the predicted values were not accurate. It often occurs in modelling, which is partly due to the number of data^50^. Also, the application of more precise data^51^ can produce better results. Moreover, the type of ML model (e.g., M5 Prime (M5P)) and the use of hybrid algorithms (e.g., random committee (RC)-RF)) may enhance the modelling accuracy^20^. In this study optimization of model parameters was accomplished through trial and error, which the use of meta-heuristic optimization algorithms (e.g., genetic algorithm (GA))^37^ can increase the efficiency of the ML model. On the other hand, since obesity is an inflammatory disease which can interfere with the results, hence, the use of variables such as body fat percentage as a more precise characteristic^12^ instead of BMI input can improve the results. Finally, it is important to consider that WBCs can also be influenced by different factors, including the menstrual cycle in females (progesterone concentration)^52^, diet, psychological stress, and environmental stress (e.g., temperature and relative humidity)^11^, which in our study were not controlled and their control may increase the accuracy of predicting WBCs using RF model.

Overall, the results of the present study as an initial step confirmed the performance of an ML model to predict the number of WBC during exercise. Furthermore, the proposed RF model in this study can help to reduce the incidence of diseases by identifying the appropriate intensity and duration of exercise.

## Conclusion

The determination of the optimal pattern of exercise training (i.e., proper intensity and duration that doesn’t suppress the immune system function) is very significant to maintain people's health. Given that, until now, no solution to this problem has been presented, hence this study was designed to develop a new method based on the RF model using the relevant and accessible variables to achieve accurate estimates of WBCs level during exercise. Results of our study demonstrated that the RF model could predict the values of WBCs during exercise using the characteristics of people (BMI, VO2 max, and WBCs values before exercise training) and the intensity and duration of exercise, which leads to achieving the optimal pattern of exercise training for people. Future studies can investigate the potential of this approach with more subjects to provide a simple RF-WBCs calculator for convenient use by athletes, non-athletes, coaches, and doctors. It should be noted our samples reflect healthy people in the age group of 18–60 years, and thus results may not be applicable to other populations (e.g., children, old people, and individuals with medical conditions).

## Data Availability

Data are available by contacting the corresponding author upon reasonable request.
